# Impact of Skeletal Muscle Mass on Physical Function and Locomotive Syndrome of Pre- and Postoperative Adult Spinal Deformity

**DOI:** 10.3390/jcm13030697

**Published:** 2024-01-25

**Authors:** Tetsuro Ohba, Kotaro Oda, Nobuki Tanaka, Goto Go, Hirotaka Haro

**Affiliations:** Department of Orthopaedic Surgery, University of Yamanashi, 1110, Shimokato, Chuo 409-3898, Yamanashi, Japan; koda@yamanashi.ac.jp (K.O.); tanakan@yamanashi.ac.jp (N.T.); ggoto@yamanashi.ac.jp (G.G.); haro@yamanashi.ac.jp (H.H.)

**Keywords:** adult spinal surgery, spinal corrective surgery, skeletal muscle mass, locomotive syndrome

## Abstract

**Background:** The purpose of this study is to identify the relationship between locomotive syndrome (LS) status, physical performance and limb and trunk skeletal muscle mass before and after surgery in adult spinal surgery (ASD) patients. **Methods:** A retrospective observational investigation of 63 consecutive patients with ASD who underwent spinal surgery was conducted. The total skeletal muscle mass of the arms and legs was considered a measure of the total appendicular skeletal muscle mass measured with whole-body dual-energy X-ray absorptiometry. All data pertaining to the physical performance tests and LS were collected preoperatively with follow-up one year postoperatively. **Results:** Gait speed, the one-leg standing test and the stand-up test were significantly improved one year after surgery compared to preoperative measurements. The lower extremity skeletal muscle mass predominantly influences physical function improvement including gait stride, one-leg standing and the stand-up test after ASD surgery. **Conclusions:** This study is the first to show that assessing lower extremity muscles prior to ASD surgery is useful in predicting postoperative recovery.

## 1. Introduction

Preparing for the coming super-aging society, the Japanese Orthopaedic Association (JOA) proposed the concept of locomotive syndrome (LS) in 2007 as a condition of reduced mobility function in an effort to decrease the number of elderly patients with musculoskeletal conditions likely to require long-term care [1]. The pathology of LS includes age-related loss of skeletal muscle mass and strength, decreased neurological activity, as well as musculoskeletal disorders such as osteoporosis, osteoarthritis, and osteoarthritis of the knee. Since then, many studies have focused on the relationship between musculoskeletal disease and LS. In the field of spinal disorders, recent studies have indicated the impact of spinal sagittal malalignment on LS in the elderly generation [2]. Furthermore, we reported that all adult spinal deformity (ASD) patients who had required surgical treatment had relatively severe LS. In an aging society, it is imperative to evaluate the LS status of patients with spinal deformities.

Sarcopenia (SP) was proposed because it treats age-related skeletal muscle deterioration as a disease concept. SP causes multiple adverse health outcomes, including physical disability, the risk of falls and fractures, decline in activities of daily living, and mortality [3]. Decreased skeletal muscle mass is considered one of the etiologies of LS and co-existence of LS and SP in the older population has been reported. Although there are differences in definition between LS and SP, there is a lot of overlap, and they can be both causes and effects of each other. Therefore, it is important to investigate the influence of skeletal muscle on LS status in ASD patients. Past studies have indicated a relationship between the skeletal muscles of the lower extremities and trunk and the compensatory mechanism of spinopelvic sagittal alignment in the elderly population with or without spinal deformity [4,5,6,7,8]. However, the impact of skeletal muscles on LS status and physical performance before and after surgery in ASD patients is largely unknown.

The purpose of the present study was to determine the relationship between LS status, physical performance and limb and trunk skeletal muscle mass before and after surgery in ASD patients.

## 2. Methods

### 2.1. Patients

This study was approved by our Institutional Review Board. We conducted a retrospective observational study of consecutive ASD patients undergoing spinal surgery. Patients indicated for thoracolumbar correction with spinal fusion who had exhausted conservative treatment options were included. Patients were 60 years of age or older and had an X-ray diagnosis of ASD defined by at least one of the following characteristics: coronal Cobb angle > 30°; C7 sagittal vertical axis (SVA) > 50 mm (between the C7 vertical line and the S1 posterior superior margin); and/or pelvic tilt (PT) > 30° (pelvis relative to femur and rest of the body (defined as the orientation of the pelvis relative to the femur and the rest of the body). Patients with lumbar spinal stenosis (LSS) had SVA < 50 mm, lumbar lordosis (LL) > 30°, and a Cobb angle < 10°. Patients with a history of adolescent idiopathic scoliosis, ankylosing spondylitis, or rounded back due to Parkinson’s disease were excluded. In total, 63 consecutive ASD patients who underwent corrective spinal surgery between April 2016 and March 2020 were included. Corrective spinal surgery was performed by two board-certified surgeons at a single institution. All data regarding physical capacity testing and LS were collected preoperatively, and patients were followed up for 1 year postoperatively.

### 2.2. Surgical Procedure of Spinal Corrective Surgery

ASD patients underwent lateral intervertebral fusion from L1-L2 or L2-L3 to L4-L5 disc level to obtain acceptable coronal and sagittal global spinal alignment. After this, patients were placed in a prone position for the posterior lumbar interbody fusion performed at the L5-S1 disc. Spinal kyphosis was corrected with cantilever correction forces with S1 bilateral single or dual iliac screws, as previously described [9], accompanied by allogenic and local autogenous bone grafts. No bone morphogenetic proteins were used.

### 2.3. Radiographic Measurements

Body composition was assessed using whole-body dual-energy X-ray absorptiometry (DXA, QDR-DELPHIW scanner DPX-NT; Hologic, Waltham, MA, USA) to measure lean body mass (LSTM), fat mass, and bone mineral content in the whole body and extremities. The sum of skeletal muscle mass in the arms and legs was considered a measure of the total appendicular skeletal muscle mass, adopting the concept that LSTM was representative of the skeletal muscle mass. Upper and lower limb LSTM (kg/m^2^) were used as the skeletal muscle index (SMI). Sarcopenia existed if the appendicular SMI was <5.46 kg/m^2^ for females and <7 kg/m^2^ for males, following guidelines in a previous study. Preoperative and one-year postoperative radiographic data were obtained from full-length frontal and lateral radiographs with the patient in an independent position with fingers resting on the clavicle. Lateral radiographic views were used to measure T5-T12 thoracolumbar kyphotic curvature; T12-S1 lumbar lordosis (LL) angles; pelvic incidence (PI); pelvic tilt (PT); sacral slope (SS); sagittal vertical axis (SVA); T1 pelvic angle (TPA), defined as the angle between the line from the center of the femoral head to the center of S1 and the line from the femoral head to the center of T1; and global tilt, predefined as the angle formed by the intersection of a line drawn from the center of C7 to the center of the sacral endplate and the second from the center of the femoral heads to the center of the sacral endplate. Kyphosis was expressed as a positive value, and lordosis was expressed as a negative value. Interobserver error was calculated from mean values of radiographic measurements made by two board-certified spine surgeons (authors 1 and 2) with >10 years of experience in spine surgery who were blinded to all patient data. The intraclass coefficient was 0.889, which means that inter-rater reliability was nearly ideal.

### 2.4. LS Screening Instrument

The Geriatric Locomotive Scale-25 (GLFS-25) is a 25-item self-administered questionnaire developed to screen for motor dysfunction in the elderly [10,11]. This consisted of questions about pain, self-care, mobility, exercise, housework, social activities, and anxiety. Responses to individual questions are rated on a scale of 0 to 4 points, which are added together to arrive at a total score and categorized as follows: no disability, not difficult to perform (0 points); mild disability, mildly difficult to perform (1 point); moderate disability, moderately difficult to perform (2 points); substantial disability, substantially difficult to perform (3 points); and severe disability, extremely difficult to perform (4 points). Total GLFS-25 scores ranged from 0 to 100 and were performed preoperatively; LS severity was based on cutoff scores: 7/100 for grade 1 LS (LS1), 16/100 for grade 2 LS (LS2), and 24/100 for grade 3 LS (LS3).

### 2.5. Physical Performance Tests

Two tests, proposed by the JOA, were used to assess LS.

(1) The two-step test: This test measures the stride length of two steps to assess overall gait ability, including lower limb strength, balance, and flexibility; it is scored by normalizing the maximum stride length of two steps by height; scores of less than 0.9, less than 1.1, less than 1.3, and greater than 1.3 on the two-step test are considered to be LS-3, LS-2, LS 1, and non-LS equivalent.
(2) The stand-up test: This test is a simple method of assessing lower extremity muscle strength by asking participants to stand up once with one or both legs from a seat at different heights (40 cm, 30 cm, 20 cm, and 10 cm). If the participants were able to stand up on one leg—both right and left legs—from the 40 cm seat and to maintain the posture for three seconds, their Locomo level was 0. The test is scored on a 0–8 point scale, with scores defined as follows: 0 (no standing), 1–4 (standing from 40 cm, 30 cm, 20 cm, and 10 cm, respectively, using both legs), 5–8 (standing from 40 cm, 30 cm, 20 cm, and 10 cm, respectively, using one leg). Scores of 0–1 point, 2 points, 3–4 points, and 5–8 points correspond to LS-3, LS-2, LS-1, and non-LS.
(3) One-legged standing time with eyes open: In this test, the time that the subject could stand for on one leg with their eyes open was measured. Measurements were taken twice for each leg, and the average time was used for analysis.

### 2.6. Handgrip Strength and Gait Speed

Grip strength was measured with a handgrip dynamometer. Both hands were tested, and the greater value was recorded as the maximum muscle strength. The average of the left and right values was recorded. Normal walking speed was measured as a measure of lower limb muscle strength. The time taken to walk 10 m down a hallway at normal walking speed was measured in seconds and manually assessed using a stopwatch to calculate normal walking speed.

### 2.7. Statistical Analysis

Mean ± SD values are reported for continuous variables or number (percentage) values used for categorical variables. Paired *t*-test and Wilcoxon signed-rank test were employed to compare mean values between two groups (pre- and postoperative), assuming normal distributions for continuous variables. Correlations between performance tests, LS screening tests, and skeletal muscle mass were determined using Pearson’s correlation coefficients after we confirmed that the variables were normally distributed using the Kolmogorov–Smirnov test.

Prism (version 9.0; GraphPad Software, La Jolla, CA, USA) was used to calculate summary statistics and perform the t tests. Asterisks indicate statistical significance (*p* < 0.05).

## 3. Results

### 3.1. Patient Population

Patient demographic information is summarized in Table 1. The mean age of subjects was 71.5 ± 6.3, all were female, and their mean BMI was 23.8 ± 3.1, mean BMD was 75.9 ± 12.7, mean SMI was 6.21 ± 10.82, and their frequency of sarcopenia defined as an appendicular SMI < 5.46 kg/m^2^ was 17/63 (26.9%).

All sagittal spinopelvic parameters were significantly improved after surgery (Table 2). The postoperative average mismatch between PI and LL was −7.3° ± 12.4°.

### 3.2. Physical Performance Tests Following Spinal Corrective Surgery

Gait speed, the one-leg standing test, and the stand-up test were significantly improved one year after surgery compared to preoperative measurements. In contrast, there was no significant improvement in gait speed, hand grip, or the two-step test one year postoperatively (Table 3).

### 3.3. Locomotive Dysfunction Following Spinal Corrective Surgery

**Figure 1:**
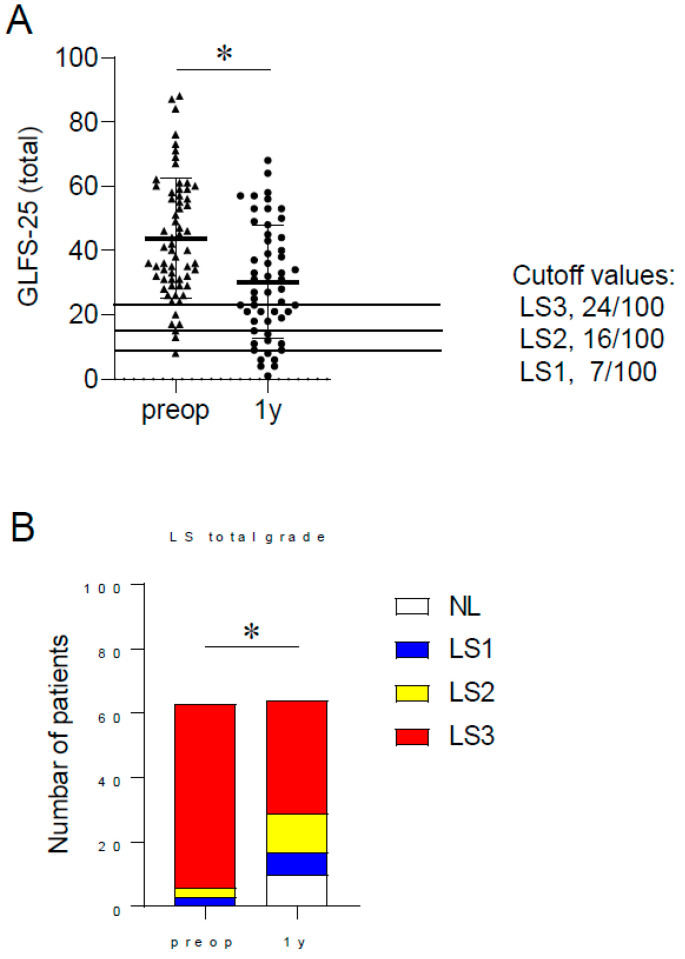
(**A**) Comparison of GLFS total scores of ASD patients preoperatively and at 1 year postoperatively (Triangle means preoperative case; Spot means postoperative case). (**B**) Comparison of severity and frequency of locomotive syndrome preoperatively and at 1 year postoperatively. LS, locomotive syndrome; NL, non-locomotive syndrome. * *p* < 0.05.

Preoperatively, all patients had LS and 57/63 (98%) were categorized as LS3. One year postoperatively, 10 patients did not have LS and the prevalence of LS3 declined to 35/63 (56%). GLFS-25 scores improved significantly one year postoperatively (preoperative; 43.7 ± 18.6, one year postoperative; 30.2 ± 17.6, Figure 1A). Both the prevalence and severity of LS had improved one year after surgery (Figure 1B).

### 3.4. Correlation between Spinopelvic Parameters, Locomotive Dysfunction, and Physical Performance Tests

Correlations for all variables were analyzed using Pearson’s correlation coefficients after checking the normal distribution of the variables.

There was no correlation between pre- and postoperative spinopelvic parameters and either locomotive dysfunction and/or physical performance tests.

### 3.5. Correlation between Skeletal Muscle Mass and Physical Performance Tests

**Figure 2:**
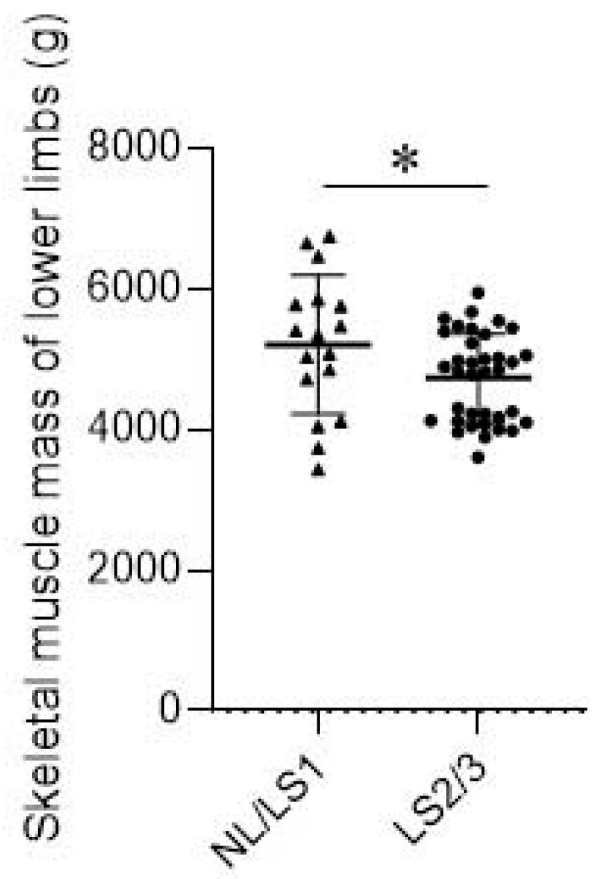
Comparison of skeletal muscle mass between non-LS/LS1 and LS2/3 group 1 year postoperatively. LS, locomotive syndrome; NL, non-locomotive syndrome. * *p* < 0.05. (Triangle means NL/LS1 cases; Spot means LS2/3 cases).

There were significant correlations between preoperative upper-arm and lower-limb skeletal muscle mass with preoperative grip strength (Table 4) but no correlation between other preoperative parameters and muscle mass. There was a significant correlation between preoperative lower limb muscle mass and postoperative gait stride (r = 0.39, *p* = 0.002), the one-leg standing test (r = 0.43, *p* = 0.006), and the stand-up test (r = 0.32, *p* = 0.031). Postoperative handgrip was significantly correlated with preoperative upper arm (r = 0.42, *p* = 0.017) and lower limb skeletal muscle mass (r = 0.47, *p* = 0.002). One year postoperatively, the NL/LS1 group had significantly greater preoperative lower extremity skeletal muscle mass than the LS2/LS3 group (*p* = 0.031, Figure 2).

## 4. Discussion

Previous studies have reported that ASD patients who have required surgical treatment have a high frequency of severe locomotive syndrome and that some improvement can be expected with surgical treatment [12,13]. Consistent with these studies, the present study revealed significant a prevalence of and severity improvements in LS in ASD patients after surgery. However, about 80% of ASD patients still have LS one year postoperatively. Therefore, it is important to identify factors that influence improvement in ASD patients after spinal corrective surgery. Unexpectedly, present data show no significant correlation between pre-and postoperative spinopelvic parameters and locomotive syndrome and physical function. In contrast, past studies indicated that spinopelvic parameters are associated with locomotive syndrome and physical function in community-dwelling middle-aged and older women who were recruited from participants performing aquatic exercise [2]. We attribute the differences in results between the studies to differences in the severity of the subjects’ spinal deformities. Overall, the subjects in this study had very poor preoperative locomotive dysfunction and physical performance; thus, we believe that the assessment items used in this study were unlikely to find the association with spinopelvic parameters.

There are many reports of correlations between lower extremity and trunk skeletal muscle mass with spinopelvic parameters, but their influence on postoperative functional outcomes is largely unknown [6,14,15]. In this study, there was no correlation between trunk muscle strength and postoperative physical function, but there was a correlation between lower extremity muscle mass and postoperative physical function of the lower extremities. To our knowledge, this is the first paper to show that lower extremity skeletal muscle mass predominantly influences physical function improvement including gait stride, one-leg standing, and the stand-up test after ASD surgery. Additionally, preoperative lower extremity skeletal muscle mass positively correlated to an improvement in LS one year postoperatively. In this study, walking speed and a 2-step test at 1 year postoperatively were not correlated with lower extremity muscle mass. We expect that this is due to the loss of flexibility of the spine and pelvis caused by spinal corrective fusion, but further study is needed.

There is a known close relationship between the spine and lower extremity function [16]. A past study indicated the association between dynamic postural change and hip extensor strength in patients with ASD who had been admitted to a medical center [17]. In contrast, our study demonstrated no correlation between preoperative physical performance and lower extremity muscle mass in ASD patients undergoing surgical treatment. Based on these results, patients with severe ASD who require surgical treatment must also use lower extremity muscle strength to compensate for poor spinopelvic alignment before surgery. Therefore, lower extremity muscle strength may be used to compensate for spinal malalignment, and lower extremity function may not be fully utilized for walking and standing ability.

This study is clinically significant because it demonstrates that lower extremity muscle strength is not correlated to preoperative physical function but is important for the recovery of physical function and/or LS after surgically improving spinopelvic parameters.

There are several limitations to this study. First, this was a retrospective study with a small sample size and psychological factors were not assessed in this study. Second, postoperative exercise therapy was not standardized. Third, the analysis was based only on skeletal muscle mass measured via DEXA. In fact, contrary to our expectations, trunk muscle mass had no effect on postoperative recovery. A future analysis of skeletal muscle mass measured via DEXA and muscle strength measurements are needed. Finally, the present study failed to evaluate the impact of other spinal conditions, such as cervical myelopathy and sarcopenia, on postoperative rehabilitation. However, this study is the first to show that assessing lower extremity muscles prior to ASD surgery is useful in predicting postoperative recovery. This study suggests that postoperative physical function may be better improved if skeletal muscle mass, measured via DEXA, and/or lower extremity muscle strength are assessed prior to ASD surgery, and a preoperative rehabilitation intervention focused on strengthening the lower extremity muscles is provided to inadequate cases.

## Figures and Tables

**Table 1:** Baseline characteristics of patients with ASD.

Variable	ASD (N = 63)
Age (y)	71.5 ± 6.3
Female/male (n)	63/0
BMI (kg/m^2^)	23.8 ± 3.1
BMD (%YAM)	75.9 ± 12.7
SMI (kg/m^2^)	6.21 ± 0.82
Sarcopenia (±)	17/46
Skeletal muscle mass (g)	
Upper arm	1606.8 ± 218.4
Trunk	16,657.3 ± 2091.6
Lower limbs	4729.3 ± 702.7

ASD, adult spinal deformity; BMI, body mass index; BMD, bone mineral density; YAM, young adult mean; SMI, skeletal muscle mass index.

**Table 2:** Preoperative and postoperative spinopelvic parameters of ASD patients.

Variable	Preoperative	Postoperative	*p* *
PT (°)	38.5 ± 12.7	18.3 ± 11.8	<0.0001
SS (°)	13.2 ± 14.8	31.9 ± 10.0	<0.0001
LL (°)	5.7 ± 25.5	56.8 ± 12.2	<0.0001
PI–LL (°)	46.0 ± 22.6	−7.3 ± 12.4	<0.0001
SVA (mm)	128.7 ± 72.8	25.2 ± 48.8	<0.0001
GT (°)	53.5 ± 16.8	17.8 ± 12.9	<0.0001
TPA (°)	44.2 ± 16.1	14.5 ± 9.5	<0.0001

ASD, adult spinal deformity; LL, lumbar lordosis; PI, pelvic incidence; PT, pelvic tilt; SS, sacral slope; SVA, sagittal vertical axis; GT, global tilt; TPA, T1 pelvic angle. Interval and ratio values are presented as the mean ± standard deviation. * *p* < 0.00001 compared with preoperative values.

**Table 3:** Physical performance tests following spinal corrective surgery.

Test	Preoperative	Postoperative, 1 Year	*p* *
Gait speed (m/s)	1.0 ± 0.28	1.1 ± 0.30	0.81
Gait stride (m)	0.52 ± 0.11	0.55 ± 0.11	0.008 *
One-leg standing test (s)	19.6 ± 2.4	22.1 ± 2.5	0.048 *
Hand grip (kg)	19.2 ± 5.3	18.6 ± 4.9	0.46
Locomotive syndrome			
Stand-up test (points)	2.9 ± 1.5	2.0 ± 1.2	0.001 *
2-step test (cm)	156.0 ± 46.8	154.4 ± 36.6	0.69

Interval and ratio values are presented as the mean ± standard deviation. * *p* < 0.05 compared with preoperative values.

**Table 4:** Correlations between skeletal muscle mass and physical performance tests.

Skeletal Muscle Mass (g)		Upper Arms	Trunk	Lower Limbs
Preoperative				
Gait speed (m/s)	r	0.13	0.14	0.04
	*p*	0.84	0.37	0.67
Gait stride (m)	r	0.19	0.21	0.14
	*p*	0.73	0.16	0.35
One-leg standing test (s)	r	−0.24	−0.18	−009
	*p*	0.34	0.22	0.51
Hand grip (kg)	r	0.38 *	0.12	0.32 *
	*p*	0.032	0.40	0.036
Stand-up test (points)	r	0.26	−0.14	0.03
	*p*	0.07	0.36	0.84
2-step test (cm)	r	0.012	−0.07	0.02
	*p*	0.40	0.66	0.89
One year after surgery				
Gait speed (m/s)	r	−0.09	−0.05	0.07
	*p*	0.55	0.97	0.65
Gait stride (m)	r	0.05	0.11	0.39 *
	*p*	0.75	0.48	0.002
One-leg standing test (s)	r	0.003	−0.19	0.43 *
	*p*	0.64	0.31	0.006
Hand grip (kg)	r	0.42 *	0.24	0.47 *
	*p*	0.017	0.12	0.002
Stand-up test (points)	r	0.02	0.11	0.32 *
	*p*	0.11	0.25	0.031
2-step test (cm)	r	−0.02	0.21	0.02
	*p*	0.90	0.41	0.87

* *p* < 0.05.

## Data Availability

where data is unavailable due to privacy or ethical restrictions.
